# Sorafenib-Loaded Cu_2−x_Se Nanoparticles Boost Photothermal–Synergistic Targeted Therapy against Hepatocellular Carcinoma

**DOI:** 10.3390/nano12183191

**Published:** 2022-09-14

**Authors:** An-Tian Huang, Jun Du, Zhi-Yong Liu, Guang-Cong Zhang, Weinire Abuduwaili, Jia-Yan Yan, Jia-Lei Sun, Ru-Chen Xu, Tao-Tao Liu, Xi-Zhong Shen, Ling Dong, Ji-Min Zhu, Yuhao Li

**Affiliations:** 1 Institute of Bismuth and Rhenium, School of Materials and Chemistry, Shanghai Collaborative Innovation Center of Energy Therapy for Tumors, University of Shanghai for Science and Technology, Shanghai 200093, China; 2 Department of Gastroenterology and Hepatology, Zhongshan Hospital of Fudan University, Shanghai 200032, China; 3 Shanghai Institute of Liver Disease, Shanghai 200032, China; 4 Department of Liver Surgery and Transplantation, Zhongshan Hospital of Fudan University, Shanghai 200032, China; 5 Key Laboratory of Medical Molecular Virology, Shanghai Medical College of Fudan University, Shanghai 200032, China

**Keywords:** sorafenib, hepatocellular carcinoma, copper(I) selenide, photothermal therapy, synergistic therapy

## Abstract

Hepatocellular carcinoma (HCC) accounts for the predominant form of liver malignancy and presents a leading cause of cancer-related death globally. Sorafenib (SOR), a first-line targeted drug for advanced HCC treatment, has a battery of untoward side effects. Photothermal therapy (PTT) has been utilized as an effective adjuvant in synergy with other approaches. However, little is known about the tumoricidal efficacy of combining SOR with PTT for HCC. Herein, a novel versatile nanoparticle, Cu_2−x_Se@SOR@PEG (CSP), that is based on a photothermal Cu_2−x_Se core and SOR for simultaneously reinforcing PTT and reducing the adverse effects of SOR was constructed. The synthesized CSP exhibited a remarkably enhanced therapeutic effect upon 808 nm laser irradiation via dampening HCC cell propagation and metastasis and propelling cell apoptosis. The intravenous administration of CSP substantially suppressed tumor growth in a xenograft tumor mouse model. It was noted that the CSP manifested low toxicity and excellent biocompatibility. Together, this work indicates a promising and versatile tool that is based on synergistic PTT and molecular-targeted therapy for HCC management.

## 1. Introduction

Hepatocellular carcinoma (HCC), the most common type of primary liver cancer; it represents the sixth leading cause of cancer and the fourth most dominant cause of cancer-related mortality worldwide [1]. Despite significant advancements in managing HCC, the 5-year survival rate for HCC patients is less than 20% [2,3]. The treatment modalities for early-stage HCC patients mostly rely on surgical resection, local ablation, and liver transplantation; however, systemic treatment is only available for of the numerous advanced HCC cases [2]. Recently, molecular-targeted therapy has been successfully approved for treating various tumors; unfortunately, most HCC cases are insensitive to these drugs as single agents [4,5]. Sorafenib (SOR), a small-molecule multi-kinase inhibitor, is the first molecular-targeted agent that was approved for the treatment of advanced HCC. However, it only prolongs the survival of patients by nearly 3 months [6,7]. Additionally, SOR often induces multiple adverse effects such as diarrhea, skin toxicity, and weight loss [7]. Therefore, it is imperative to develop a safe and effective strategy for HCC management.

It is well characterized that photosensitizer-mediated photothermal therapy (PTT) has outstanding advantages, such as minimal invasiveness, high specificity, and low toxicity [8,9,10]. In particular, when the near-infrared (NIR) biological window (700–1400 nm) is harnessed to irradiate the tumor regions, the laser excites the photothermal conversion of the nanoparticles that are aggregated in the tumor-specific sites so as to convert light energy into heat, which directly instigates tumor cell death and lessens the self-heating of healthy tissues or organs [11,12,13,14,15]. Cu_2−x_Se nanoparticles have excellent photothermal conversion properties and biocompatibility and they could be utilized as a drug carrier or therapeutic reagent [16,17,18]. Secondly, an ultrasmall Cu_2−x_Se nanoparticle is less likely to be rapidly recognized and cleared by phagocytes and the reticuloendothelial system, thus circulating in the blood for a longer time and passively accumulating within tumors through the enhanced permeability and retention effect [19,20,21,22,23]. Thirdly, despite diverse preparation methods, the Cu_2−x_Se nanoparticle shows uncomplicated synthesis, excellent reproduction, and great dispersity for bio-applications [17,19,24].

In order to enable a Cu_2−x_Se nanoparticle to be applied in bio-applications, it is essential to make a surface modification [17,24,25,26,27,28]. Hessel et al. prepared Cu_2−x_Se nanoparticles with a size of 16 nm through a method of hot injection [17]. After modification by an amphiphilic polymer, this method proved to be a fruitful way to obtain a kind of photothermal transduction reagent [17]. Zhang et al., prepared Cu_2−x_Se nanocrystals with a size of ca. 7.6 nm by using a similar method [29]. After being modified by a phospholipid, they used the nanocrystals as a contrast agent for the in vivo PA imaging of a sentinel lymph node [29]. Zhang et al. reported a simple aqueous route to prepare water-soluble and biocompatible ultrasmall Cu_2−x_Se nanoparticles for the multimodal imaging of cancer [19]. Therefore, the potential applications of Cu_2−x_Se nanoparticles in biology are fantastic [30,31,32]. In order to prolong the survival of HCC patients and improve their survival quality, we sought to reduce the side effects of the targeted drug SOR and synergistically enhance the anti-HCC effects by combining the imperfect targeted drug and photothermal therapy with definitive efficacy.

**Scheme 1:**
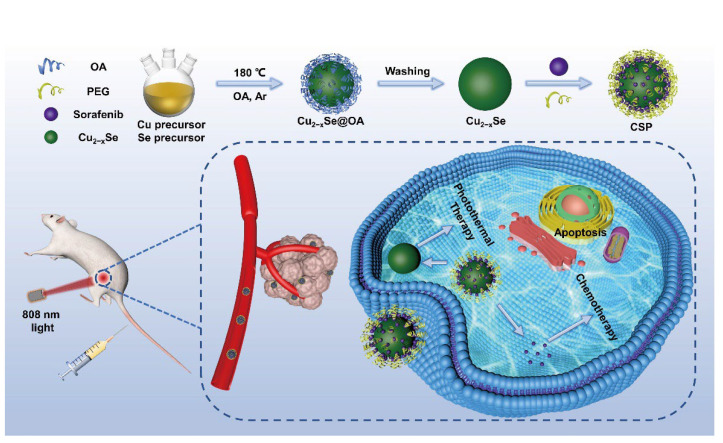
Schematic diagram of the preparation of CSP and its synergistic photothermal-chemotherapy against hepatocellular carcinoma.

Herein, we designed and prepared a novel nanoparticle, Cu_2−x_Se@SOR@PEG (CSP), that comprises a photothermal Cu_2−x_Se core, a molecular-targeted drug SOR, and amphipathic polyethylene glycol (PEG) in order to enhance the PTT effect and to reduce the side effects of SOR for HCC treatment (Scheme 1). The in vitro and in vivo experiments showed that, when employing the synergistic effects of PTT and chemotherapy, the CSP nanoparticles, upon laser irradiation, significantly inhibited the HCC cells’ proliferation and metastasis and reversed the tumor microenvironment, thereby ablating all tumor growth. This provides a promising avenue for ameliorating the efficacy of the chemotherapeutic agent SOR in the treatment of liver cancer.

## 2. Experimental Section

### 2.1. Materials

All of the chemicals were directly attained from commercial sources without any further purification. Selenium powder (Se, 99.99%) was purchased from Adamas-beta (Adamas-beta, Shanghai, China), while cuprous chloride (CuCl, 99.999%) was acquired from Alfa Aesar (Alfa Aesar, Ward Hill, MA, USA). Oleic acid (OA, 90%) and oleylamine (OAm, 70%) were obtained from Sigma-Aldrich (Merck, Burlington, NJ, USA). Sorafenib (SOR, ≥99%) was purchased from Aladdin (Aladdin, Shanghai, China).

### 2.2. Characterization

Transmission electron microscopy (TEM) images and elemental mapping were measured on an FEI Tecnai F20 (FEI, Columbia, SC, USA). X-ray diffraction (XRD) was acquired from the Bruker D8 instrument (Bruker, Saarbruecken, Germany). X-ray photoelectron spectra (XPS) were obtained from Thermo Scientific K-Alpha (Thermo Fisher Scientific, Waltham, MA, USA). The hydrodynamic size of the sample was attained from a nanoparticle size analyzer (Zetasizer Nano ZS90, Malvern, Malvern, UK). UV–vis absorption spectra were measured by a spectrophotometer (UV–1900, Shimadzu, Kyoto, Japan). The photothermal properties of the samples were recorded by an infrared imaging device (Nicolet 380, Madison, WI, USA).

### 2.3. Synthesis of Cu_2−x_Se Nanoparticles

The Cu_2−x_Se nanoparticles were synthesized as per the method of a previously reported protocol with some modifications [17]. To begin with, selenium powder (158.0 mg, 2 mmol) was added to a reaction flask with 20 mL of OA. After 30 min of evacuation at 100 °C, argon gas was injected into the reaction system continuously for the purpose of insulating the solution from the air. Following this, the temperature was risen to 310 °C. After stirring for about 10 min at this temperature, the solution was cooled to 60 °C in order to obtain the Se precursor. Subsequently, CuCl (100.0 mg, 1 mmol) was added to another reaction flask with a mixture of 8 mL of OA and 12 mL of OAm. After 30 min of evacuation at 100 °C, a steady flow of argon was continuously injected into the reaction system. Then, the temperature was risen to 230 °C and the mixture was kept for about 10 min at this temperature in order to afford the Cu precursor. Finally, the Se precursor was injected into the reaction flask with the Cu precursor for 10 min with stirring at 230 °C. After cooling to room temperature and washing with ethanol, the Cu_2−x_Se nanomaterial was synthesized.

### 2.4. Fabrication of CSP

The poly(maleic anhydride-*alt*-1-octadecene)-poly(ethylene glycol) methyl ethers) (PMHC_18_-mPEG) was synthesized following a previously documented protocol [33]. The Cu_2−x_Se nanoparticle (5.0 mg), sorafenib (0.5 mg), and PMHC_18_-mPEG (15.0 mg) were added into 15 mL of chloroform and stirred for 30 min at room temperature. Then, the solvent was removed by rotary evaporation. After drying for about an hour, the final composite was dispersed into deionized water.

### 2.5. Photothermal Properties of CSP

The photothermal properties of the CSP were measured by a thermal imager (FLIR E40, Teledyne FLIR, Wilsonville, OR, USA) under 808 nm light irradiation. The composite CSP was dispersed in water at different concentrations and irradiated with an 808 nm laser under different power densities. To begin with, the concentration of the CSP was fixed (100 μg mL^−1^) and the irradiation’s power density increased from 0.6 to 1.2 W cm^−2^. Then, the power density was fixed (1.0 W cm^−2^) and the concentration of the CSP increased from 25 to 200 μg mL^−1^.

Secondly, the CSP solution (100 μg mL^−1^) was irradiated with 808 nm light (1.0 W cm^−2^) for 10 min, we switched off the light until the solution cooled to room temperature, and the thermal performance of pure water was compared under the same conditions. Thirdly, the photothermal stability of the CSP (100 μg mL^−1^) was attained by turning on (10 min) and off (10 min) light (1.0 W cm^−2^) five times. Finally, the photothermal conversion efficiency could be calculated by the following reported method [34].

Formula (1) could then be used to calculate the photothermal conversion efficiency:

(1) $$\eta =\frac{hA\left(T_{max}-T_{amb}\right)-Q_{0}}{I\left(1-10^{-A_{\lambda }}\right)}$$

where *h* is the heat transfer coefficient and *A* is the surface area, *I* is the laser power, *A_λ_* is the absorbance of CSP dispersed in water at 808 nm excitation wavelength, and *Q*_0_ is the heat that was absorbed by the solvent. The value of *hA* could then be determined by applying the following formula:

(2) $$t=-\tau_{s}\cdot ln\left(\theta \right)$$

where *t* is cooling time and θ is calculated by the following formulas:

(3) $$\theta =\frac{T\left(t\right)-T_{surr}}{T_{max}-T_{surr}}$$

(4) $$\tau_{s}=\frac{m_{D}C_{D}}{hA}$$

where *τ_s_* is the time constant and *m_D_* and *C_D_* are the mass and specific heat capacity of deionized water that was used as a solvent, respectively.

### 2.6. Cell Culture

Human HCC cell lines, MHCC-97H and Huh-7, were cultured in DMEM (Wisent, Shanghai, China) and supplemented with 10% fetal bovine serum (FBS, Gibico, Waltham, MA, USA) and 1% penicillin/streptomycin. Immortalized hepatic epithelial THEL-2 cells were reared in Bronchiolar Epithelium Growth Medium (BEGM) (Meisen, Jinhua, China) and regarded as normal hepatocytes. All of the cultures were maintained in an incubator at 37 °C with a humidified 5% CO_2_ atmosphere.

### 2.7. Cytotoxicity Assessment

Cancerous or normal cells were seeded into 96-well plates at a density of 1 × 10^4^ cells per well and then incubated. The corresponding media carrying the CSP nanoparticles at different concentrations (0, 5, 10, 20, 35, and 50 μg mL^−1^) were added into the wells and co-cultured with the cells for 24 h. The cells were then irradiated with an 808 nm laser at a density of 1 W cm^−2^ for 2 min. After being cultured for another 24 h or 48 h, the cells’ viability was evaluated by using the cell counting kit-8 (CCK-8, Beyotime, Shanghai, China) according to the manufacturer’s instructions. The absorbance at 450 nm of each well was recorded by the micro-plate reader.

### 2.8. Colony Formation and Wound Healing Assays

In order to determine the effects of the different formulations on the proliferation and metastasis of the HCC cells in vitro, the logarithmic HCC cells were seeded in 6-well plates at a density of 1 × 10^3^ per well and grown overnight. After cell attachment, different formulations were added to the wells. The cells were then irradiated with an 808 nm laser at a density of 1 W cm^−2^ for 2 min and grown for 2 consecutive weeks. The cells were washed twice with PBS, fixed with 4% paraformaldehyde for 20 min, and stained with 0.1% crystal violet. After that, the 6-well plate was rinsed thoroughly with tap water, air-dried at room temperature, and photographed. The number of cell colonies was counted via the software ImageJ v.1.53t.

Furthermore, wound healing was performed as has been previously described [35]. In brief, after the wells grew full of cells, a cell scratch was generated by utilizing a sterile tip and the sloughing cells were removed using PBS. Subsequently, 3% FBS DMEM, including various formulations, was added to the wells. After the wells had been incubated for 48 h, the wound closure was photographed by an inverted microscope (Olympus, Tokyo, Japan). The scratch areas were calculated by the software ImageJ v.1.53t.

### 2.9. Dead/Live Cell Staining

In order to determine the antitumor performance of the CSP nanoparticles, we tested the status of the live and dead cells using a dead/live cell staining kit (Beyotime, Shanghai, China) according to the manufacturer’s instructions. In short, the cells were seeded into 24-well plates and cultivated in an incubator. After cell attachment, the cells were incubated with different formulations for 24 h. For the laser treatment groups, the cells were irradiated with an 808 nm laser at a density of 1 W cm^−2^ for 2 min. The cells were flushed twice with PBS. Following this, a Calcein-AM–PI working solution was prepared, added to each well, and incubated with the cells for 30 min in dark conditions. After that, the cells were photographed by a fluorescent microscope (Olympus, Tokyo, Japan). Viable cells are stained green by the Calcein-AM, whereas dying cells are usually red fluorescent from the PI.

### 2.10. Apoptotic Assays

Apoptotic cells were detected through an apoptosis detection kit (Beyotime, Shanghai, China) following the manufacturer’s instructions. Briefly, cells were seeded into 6-well plates. After cell attachment, the cultured cells were incubated with different formulations for 24 h. For the CSP plus laser group, the cells were irradiated for 2 min with an 808 nm laser at a density of 1 W cm^−2^. The cells were cultured for another 24 h, after which they were collected by centrifugation. Afterward, the cells were washed twice with ice-cold PBS and then re-suspended in a 200 μL binding buffer. The cells were stained with 5 µL Annexin V-FITC and 10 µL propidium iodide (PI) for 30 min at 37 °C in dark conditions. The components of the apoptotic cells were assayed and analyzed using a flow device (BD, San Jose, CA, USA).

### 2.11. Immunoblot

For protein extraction from the cell samples that were treated with different formulations, the cell pellets were re-suspended in 100 μL RIPA buffer (Beyotime, Shanghai, China) and an equal volume of 2 × SDS loading buffer was added. The tubes were boiled at 100 °C for 10 min and then centrifuged at 4 °C at 12,000 rpm for 10 min. Subsequently, the supernatants were transferred into clean tubes. A Western blot was performed as has been previously described [36], with a few exceptions. In brief, the supernatant proteins were separated utilizing SDS-PAGE gel and then moved into the PVDF membrane. The membranes were blocked with 5% skim milk in TBS-T buffer. The PVDF membrane was incubated with the primary and HRP-conjugated secondary antibodies in turn. The protein bands were assayed and photographed using the enhanced chemiluminescence (ECL) reagent and gel imaging system (Tanon, Shanghai, China).

### 2.12. In Vivo Co-Therapy against Tumor

The 4-week-old BALB/c nude mice that were used in this experiment were obtained from the Model Animal Research Center of Nanjing University. The animal protocol was approved by the Ethics Committee of Zhongshan Hospital, Fudan University (RZ202105060017). All of the mice were conventionally bred and maintained in the specific pathogen-free (SPF) barrier facility at the Department of Laboratory Animal Science at Fudan University (SYXK-2020-0032). The mice were housed under a 12 h light–dark cycle, with ad libitum access to drinking water. The Human HCC xenograft tumor model was constructed by subcutaneous (s.c.) injection of Huh-7 cells into the flank of each mouse. When the tumor size reached about 5 mm, the mice were randomly divided into six groups with six mice per group and then intravenously (i.v.) injected with 100 µL of the various formulations for a total of four times on days 21, 24, 27, and 30. For the laser treatment groups, the tumors were irradiated for 2 min with an 808 nm laser at a density of 1 W cm^−2^, two hours after the injection. On day 35, the mice were anesthetized with pentobarbital sodium and killed. The tumor size was measured using a caliper and the tumor volume was calculated according to the formula (a × b^2^)/2, in which a and b individually denote the long and short diameter of the tumor. Concordantly, the tumor weight was also measured as a proxy of the efficacy of the nanoparticle CSP.

### 2.13. Hematoxylin and Eosin (HE) Staining and Immunohistochemistry (IHC)

HE staining and IHC were carried out as have been previously described [37]. Briefly, after the indicated treatments, the tumors or main organs (heart, liver, spleen, lung, and kidney) were excised, fixed in 4% paraformaldehyde, embedded in paraffin, and then cut into a series of slices. These paraffin slices were baked at 60 °C for 2 h, dewaxed in xylene, and then dehydrated in gradient ethanol. For the HE staining process, the slices were dyed with hematoxylin and eosin. In addition, the slices for the IHC were separately incubated with primary antibodies against Ki-67 and VEGFA (Powerful Biology, Beijing, China) at 4 °C overnight and then treated with corresponding secondary antibodies at 37 °C for 1 h. The sections were developed using diaminobenzidine, counterstained using hematoxylin, and analyzed with the software ImageJ v.1.53t.

### 2.14. Hematologic Assays

Whole blood was collected from the BALB/c nude mice and heparin sodium was added to it as an anticoagulant. Erythrocytes were harvested by centrifugation, rinsed three times with PBS, and diluted ten times with PBS. Various concentrations of CSP were added to the cell suspensions. PBS was considered a negative control and water as a positive control. The mixes were incubated at 37 °C for 3 h and spun at 3000 rpm for 10 min. The absorbance of the supernatants was detected at 545 nm by a spectrophotometer. In addition, blood biochemical indicators such as alanine aminotransferase (ALT), aspartate aminotransferase (AST), blood urea nitrogen (BUN), and serum creatinine (SCR) were tested for the assessment of liver or kidney function.

### 2.15. Statistics

The in vitro and in vivo experimental data are shown as mean ± standard deviation (SD). A two-tailed, non-paired Student’s *t*-test was used for the comparison between two groups and one-way analysis of variance (ANOVA) was used for multiple group comparison. Statistical significance is indicated in the figures as follows: ns denotes no significance; * denotes *p* < 0.05, ** denotes *p* < 0.01, and *** denotes *p* < 0.001. All of the experiments were repeated at least three times and yielded consistent results.

## 3. Results and Discussion

### 3.1. Preparation and Characterization of CSP

**Figure 1:**
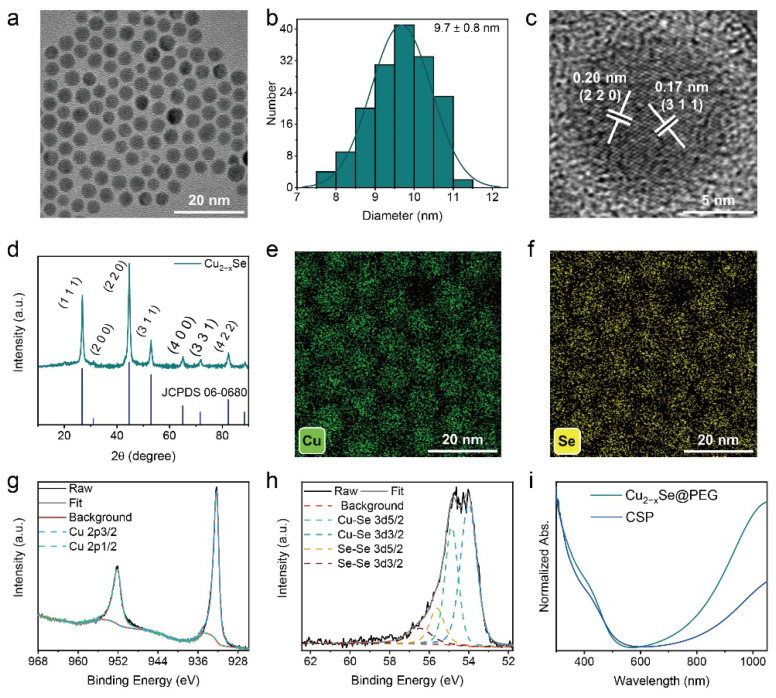
Morphology and structure of Cu_2−x_Se. (**a**) TEM image, (**b**) particle size distribution, (**c**) HR-TEM image, and (**d**) XRD of Cu_2−x_Se. EDS mapping of (**e**) Cu and (**f**) Se. HR-XPS of (**g**) Cu and (**h**) Se. (**i**) Absorption spectra of Cu_2−x_Se@PEG and CSP.

In this study, the CSP was synthesized by a two-step procedure (Scheme 1). The Cu_2−x_Se nanoparticles were firstly synthesized by a method of thermal injection. Subsequently, amphiphilic PEG was assembled into hydrophobic Cu_2−x_Se so as to form a hydrophobic cavity that SOR could be loaded into in order to form a CSP composite through hydrophobic interaction. The transmission electron microscope (TEM) image of the Cu_2−x_Se nanoparticles showed a spheroid with uniform particle morphology (Figure 1a). According to the rendered statistics, the average particle size was approximately 9.7 ± 0.8 nm with a small size distribution (Figure 1b). The high-resolution TEM (HR-TEM) image showed the lattice spacing of Cu_2−x_Se. Two lattice spacings of 0.20 nm and 0.17 nm were assigned to the (220) planes and (311) planes, respectively (Figure 1c). The X-ray diffraction (XRD) pattern was well matched with the standard diffraction card of Cu_2−x_Se (JCPDS card No. 06-0680), indicating the high purity of the synthesized Cu_2−x_Se (Figure 1d). Furthermore, the elemental EDS mapping results indicated that Cu and Se were homogeneously distributed in particles and that the atomic ratio of Cu and Se was close to 2:1, indicating a copper defective type Cu_2−x_Se (Figure 1e,f and Figure S1).

In order to discuss the chemical composition and states of the Cu_2−x_Se, X-ray photoelectron spectroscopy (XPS) was performed (Figure 1g,h and Figure S2). In the high-resolution spectrum (HR-XPS) of Cu, two strong peaks at 932.28 eV and 952.18 eV could be attributed to Cu 2p_3/2_ and Cu 2p_1/2,_ respectively; the binding energy difference between the two peaks was about 20 eV, revealing that the Cu was almost in a valence state of +1. In the HR-XPS of Se, two strong peaks were monitored at 54.03 eV and 54.88 eV and the binding energy difference between the two peaks was about 0.85 eV, which could be assigned to the bond of Cu–Se. Two weak peaks at 55.63 eV and 56.48 eV (the binding energy difference between which was also about 0.85 eV) could be ascribed to the bond of Se–Se. The excessive selenium from this bond might suggest the Cu deficiency in the Cu_2−x_Se. The XPS results demonstrated that the element in the Cu_2−x_Se was ionic and had a valence state of +1 and −2 for Cu and Se.

By modifying the surface of the Cu_2−x_Se with amphiphilic PEG (Figure S3), it was possible to form a hydrophobic cavity not only for loading SOR but also for improving water dispersibility. The UV-vis absorption spectra showed that the light absorption properties of PEG-modified Cu_2−x_Se (named Cu_2−x_Se@PEG, without loading SOR) did not change significantly before and after loading SOR and that it had better light absorption ability in the NIR region (Figure 1i). In addition, the hydrated particle size of the Cu_2−x_Se@PEG, as determined by dynamic light scattering, was 170 nm (Figure S4). The increase in size might be caused by an enlarged hydrophobic cavity after loading SOR or the formation of a hydrated layer between the PEG and water. Furthermore, the Cu_2−x_Se@PEG was well dispersed in the physiological buffer solutions such as phosphate buffer solution (PBS) and Dulbecco’s modified eagle medium (DMEM) and can be further used in subsequent experiments.

### 3.2. Photothermal Properties of Cu_2−x_Se@PEG

**Figure 2:**
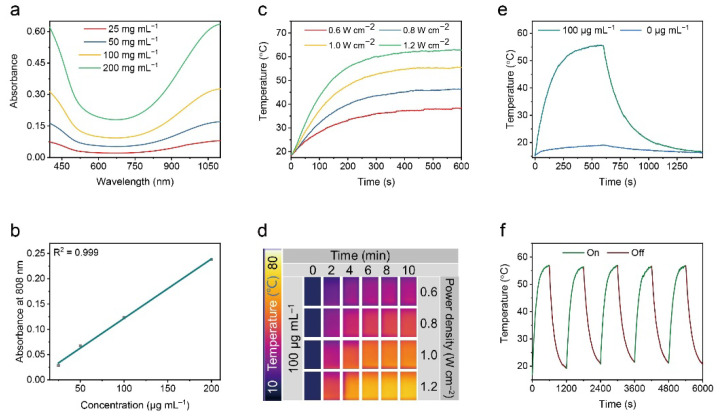
Photothermal properties of Cu_2__−x_Se@PEG in water. (**a**) Absorption spectra of Cu_2__−x_Se@PEG with different concentrations. (**b**) The absorbance at 808 nm from (**a**) variation of temperature (**c**) and thermal imaging (**d**) with prolonged irradiation time under 808 nm laser irradiation at different power densities. (**e**) Photothermal curves of Cu_2__−x_Se@PEG and water under 808 nm laser irradiation with a power density of 1.0 W cm^−2^ for 10 min and then cooling naturally. (**f**) Photothermal stability was attained from several irradiation cycles (808 nm laser on/off, 1.0 W cm^−2^).

Due to the strong near-infrared light absorption properties of Cu_2−x_Se@PEG, it can be used for photothermal conversion. The light-absorbing ability of the Cu_2−x_Se@PEG gradually increased with its concentration (Figure 2a). The absorbance of the Cu_2−x_Se@PEG at 808 nm had a positive correlation with the concentration and conformed to the Beer–Lambert law, which is beneficial in exploring the photothermal conversion ability of Cu_2−x_Se@PEG (Figure 2b). Since water absorbs light at 808 nm weakly and exhibits better biosafety, we chose this wavelength to excite the Cu_2−x_Se@PEG. When fixed at the concentration of 100 μg mL^−1^, the Cu_2−x_Se@PEG exhibited a better thermal effect with prolonged laser irradiation time and its heating rate and temperature increased with the excitation light’s power (Figure 2c,d). At an excitation light power of 1.0 W cm^−2^, the temperature increased by 40.3 °C. In addition, the thermal effect of the Cu_2−x_Se@PEG also showed a positive correlation with the concentration (Figure S5). These results indicate that the photothermal effect can be co-regulated by the light’s power density and the concentration of the Cu_2−x_Se@PEG. In contrast, under irradiation of the same power density, the thermal effect of water at 808 nm is weak (Figure 2e). After calculation, the photothermal conversion efficiency of the Cu_2−x_Se@PEG was found to be 27.6%. Finally, after 5 cycles of 808 nm laser illumination, the thermal effect that was generated by the Cu_2−x_Se@PEG remained unchanged in the stability test, suggesting excellent thermal stability (Figure 2f). All of these results suggest that Cu_2−x_Se@PEG is appropriate to be applied for tumor photothermal therapy.

### 3.3. Cytotoxicity of CSP on HCC Cells In Vitro

**Figure 3:**
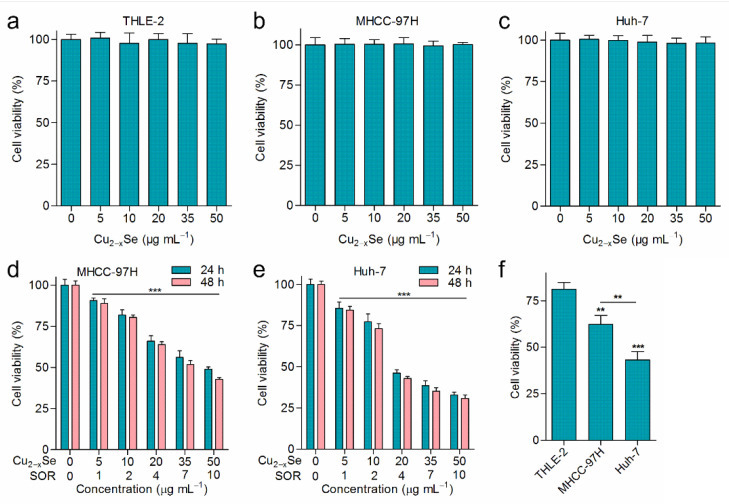
Cytotoxicity of nanoparticle Cu_2−x_Se@PEG and CSP against normal and HCC cells. Viability of THLE-2 (**a**), MHCC-97H (**b**), and Huh-7 (**c**) cells incubated with different concentrations of Cu_2−x_Se for 24 h. Viability of MHCC-97H (**d**) and Huh-7 (**e**) cells incubated with various concentrations of CSP nanoparticles for 24 h or 48 h, and then irradiated with 808 nm laser. (**f**) Sensitivity of normal hepatocyte and HCC cells to CSP nanoparticles by 808 nm laser irradiation. ns denotes no significance; * denotes *p* < 0.05, ** denotes *p* < 0.01, and *** denotes *p* < 0.001.

It is well established that most HCC cases are unresponsive to SOR and develop resistance after treatment [5]. In order to address the limited benefits of SOR for HCC, we chose the photosensitizer Cu_2−x_Se as a delivery carrier so as to alleviate the apparent adverse events of SOR. The cytotoxicity of the as-prepared Cu_2−x_Se@PEG on normal hepatic and HCC cells was tested by the CCK-8. In the absence of 808 nm laser irradiation, the various concentrations of Cu_2−x_Se@PEG that were incubated with THLE-2 cells did not exhibit obvious cytotoxicity after 24 h of cultivation (Figure 3a). Moreover, we found that cell viabilities were greater than 90% when the different concentrations of Cu_2−x_Se@PEG were incubated separately with the MHCC-97H and Huh-7 cells for 24 h (Figure 3b,c), indicating that Cu_2−x_Se@PEG had excellent biocompatibility.

Subsequently, Cu_2−x_Se@PEG was harnessed as a SOR carrier in order to mitigate its adverse effects. The CSP nanoparticles that were synthesized with Cu_2−x_Se@PEG and SOR at a 5 to 1 mass ratio were evaluated for their cytotoxicity in vitro. Various concentrations of the CSP nanoparticles were then added to a 96-well plate and incubated separately with MHCC-97H and Huh-7 cells. Upon 808 nm laser exposure, the level of cytotoxicity was augmented significantly with each increasing concentration of the CSP nanoparticles in the MHCC-97H cells (Figure 3d). Furthermore, the cytotoxicity of the CSP nanoparticles to Huh-7 cells showed the same pattern as that of the MHCC-97H cells (Figure 3e). Notably, the viability exhibited a clear inflection point in MHCC-97H or Huh-7 cells and then started to slow down when the concentration of Cu_2−x_Se increased to reach 20 μg mL^−1^. As a consequence, 20 μg mL^−1^ of Cu_2−x_Se in the CSP nanoparticles was used for subsequent experiments. We also compared the sensitivity of various HCC cells to the CSP nanoparticles by applying 808 nm laser irradiation. The results revealed that the Huh-7 cells were more sensitive to the CSP nanoparticles as compared with the MHCC-97H cells and that the viability of the Huh-7 cells declined to 43.18% (Figure 3f). The results, therefore, indicate that combined Cu_2−x_Se and SOR therapy could alleviate the untoward side effects of SOR.

### 3.4. Antitumor Effectiveness of CSP via Repressing Cell Proliferation and Metastasis In Vitro

**Figure 4:**
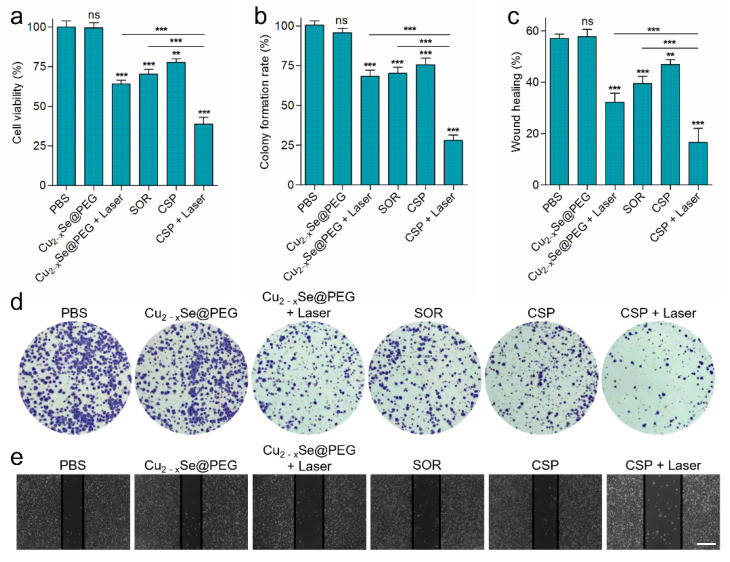
Antitumor effect via repressing HCC cell proliferation in vitro. (**a**) Cytotoxicity of PBS, Cu_2−x_Se@PEG, Cu_2−x_Se@PEG plus Laser, SOR, CSP, and CSP plus laser in Huh-7 cells. Colony formation (**b**) and statistical analysis (**d**) of Huh-7 cells treated with various formulations. Wound healing (**c**) and statistical analysis (**e**) of Huh-7 cells treated with various formulations. Scale bar, 200 μm. ns denotes no significance; * denotes *p* < 0.05, ** denotes *p* < 0.01, and *** denotes *p* < 0.001.

In order to confirm the synergistic effect of the CSP nanoparticles, the HCC cells were challenged with different treatments including PBS, Cu_2−x_Se@PEG, Cu_2−x_Se@PEG plus laser, SOR, CSP, and CSP plus laser. After being treated with CSP plus laser for 48 h, the cytotoxicity in the Huh-7 cells reached over 60% and the viability loss was significantly lower than that of Cu_2−x_Se@PEG plus laser or SOR alone (Figure 4a). In addition, we also found that the Huh-7 cells were susceptible to SOR compared to their reaction to CSP without laser irradiation. This effect might be attributed to the slow release of SOR. The data have illustrated that the CSP nanoparticles, upon laser irradiation, could exert antitumor activities through the combination of photothermal and chemotherapeutic effects.

Next, we further evaluated the method’s ability to kill tumor cells by using a clonogenic assay. As is shown in Figure 4b,d, there were 68.37% and 70.21% colony formations of the Huh-7 cells after their treatment with Cu_2−x_Se@PEG plus laser or SOR alone, respectively, while only 27.94% of the cells survived in the CSP plus laser group. The results suggested that the CSP nanoparticles had the potential to act as photothermal drugs. Furthermore, we measured the cytotoxicity of the CSP nanoparticles on the tumor cells by the use of a scratch experiment. It was found that the healing rate in the CSP plus laser group was substantially reduced compared with that of the Cu_2−x_Se@PEG plus laser or SOR alone groups in Huh-7 cells, while the healing rate in the Cu_2−x_Se group was not significantly constrained as compared with the control PBS group (Figure 4c,e). The results revealed that CSP nanoparticles combined with photothermal and chemotherapeutic therapy could conspicuously repress HCC cell proliferation. Based on these results, a combinational therapy using CSP plus laser could effectively decrease the SOR dose, thus alleviating the side effects of SOR.

### 3.5. Antitumor Effectiveness of CSP via Inducing Cell Apoptosis In Vitro

**Figure 5:**
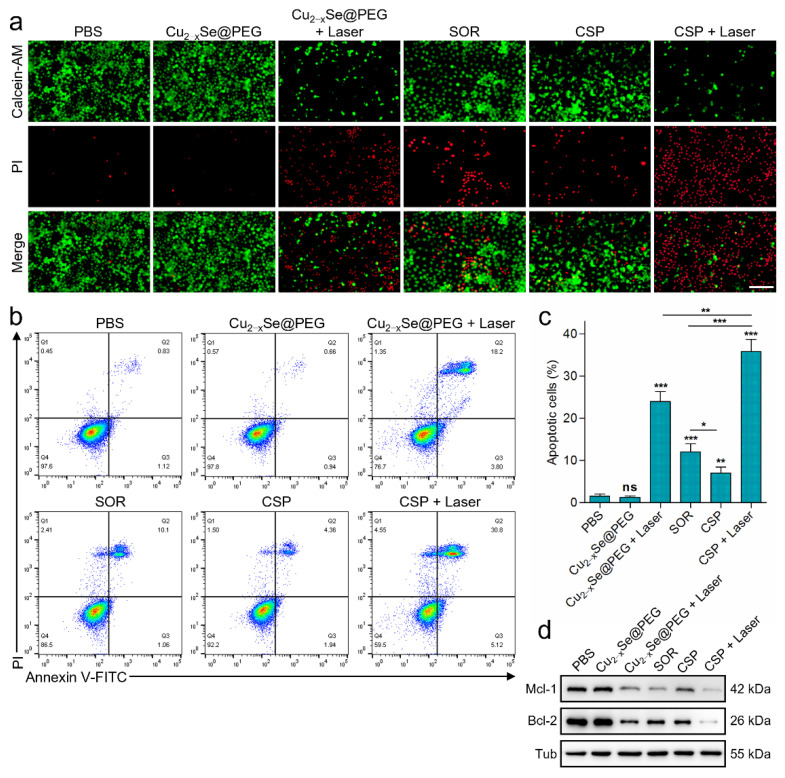
Death of HCC cells triggered by various formulations. (**a**) Representative fluorescent images of Huh-7 cells stained with Calcein-AM and PI following different formulations: PBS, Cu_2−x_Se@PEG, Cu_2−x_Se@PEG plus laser, SOR, CSP, and CSP plus laser. Live and dead cells as shown by green and red signals. The scale bar represents 200 μm. Apoptosis (**b**) and statistical analysis (**c**) of Huh-7 cells treated with various formulations. (**d**) Western blot analysis of Mcl-1 and Bcl-2 in Huh-7 cells treated with different formulations. The protein β-tubulin (Tub) was used as a loading control for protein level assays. ns denotes no significance; * denotes *p* < 0.05, ** denotes *p* < 0.01, and *** denotes *p* < 0.001.

In order to further corroborate the anti-tumor performance of the CSP nanoparticles after laser irradiation, we co-stained the Huh-7 cells with calcein-acetoxymethyl easter (Calcein-AM) and propidium iodide (PI) so as to distinguish the live (green fluorescence) and dead (red signal) cells. No apparent dying cells were observed in the Huh-7 cells that were treated with Cu_2−x_Se@PEG alone, which was a result that was similar to that of the control group without any treatment (Figure 5a). The cells that were incubated with CSP followed by laser irradiation generated more widespread and intense red fluorescence than the Cu_2−x_Se@PEG plus laser or SOR alone groups, suggesting that the CSP plus laser group had good killing effects on tumor cells. Multiple pieces of evidence have proposed that apoptosis is the primary form of regulated cell death for tumor cells when they are treated with different agents; although, necroptosis, pyroptosis, and ferroptosis have important roles in various treatment contexts [38,39,40,41]. Therefore, the tumor-killing effect of CSP plus laser irradiation was further evaluated using flow cytometry. After incubation with the mock PBS or Cu_2−x_Se@PEG alone, the apoptotic rate of the Huh-7 cells was individually 1.56% and 1.23%, further indicating the low toxicity of the as-synthesized Cu_2−x_Se@PEG nanoparticles. For contrast, the apoptotic rate of the Huh-7 cells was 23.98% and 12.05% after treatment with Cu_2−x_Se@PEG plus laser and SOR alone, respectively; while the CSP plus laser group exhibited the best killing effect on the Huh-7 cells with an increased apoptotic rate of 35.83% (Figure 5b,c).

It is well characterized that SOR accelerates cell apoptosis by down-regulating the expression of the anti-apoptotic protein Mcl-1 and that targeting Bcl-2 signaling can augment sorafenib-induced apoptosis in HCC [42,43,44]. Additionally, photosensitizer-based photothermal therapy has been extensively used for improving the potency of systemic treatment through enhancing targeted agent-provoked apoptosis [45,46]. Thereafter, we detected the expression of apoptosis-associated proteins using a Western blot test. The expression of anti-apoptotic proteins (including Mcl-1 and Bcl-2) in the CSP plus laser group was reduced compared to that of the other treatment groups (Figure 5d). These data demonstrated that the traditional apoptotic pathway might mediate the cytotoxicity of the CSP nanoparticles via curtailing the expression of the anti-apoptotic proteins in HCC cells. Taken together, these results reveal that photothermal therapy enhances the effect of SOR on HCC cells by regulating cell proliferation and apoptosis, providing a potential strategy to kill tumor cells.

### 3.6. In Vivo Synergistic Tumoricidal Activity of CSP Nanoparticles

**Figure 6:**
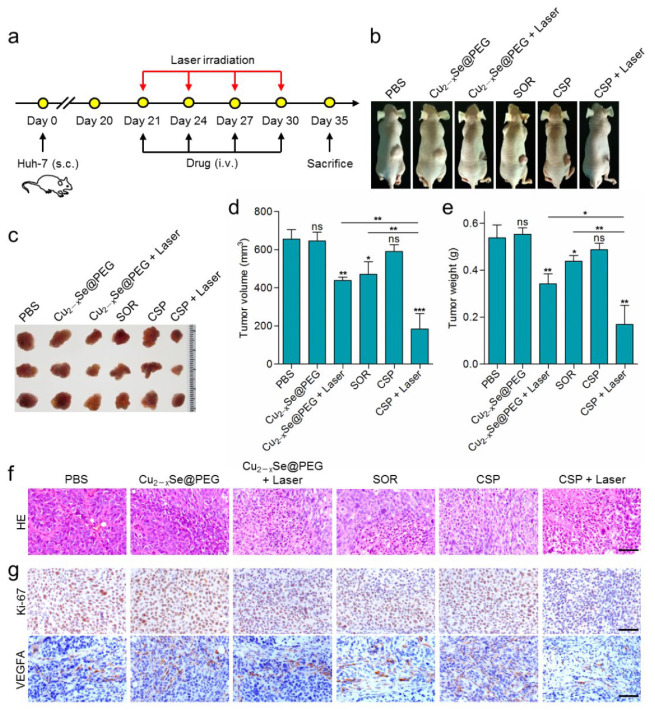
Synergistic targeted and photothermal therapy using CSP in vivo. (**a**) Schematic of model construction and HCC treatment using a xenograft tumor model in vivo. Representative photographs of mice (**b**) and dissected tumors (**c**) after 35 days of administration of various formulations. Tumor volume (**d**) and tumor weight (**e**) of mice on day 35. (**f**) HE-stained tumor slices from six groups of mice on day 35. (**g**) IHC staining of Ki-67 and VEGFA in tumor tissues. Scale bar, 50 μm. ns denotes no significance; * denotes *p* < 0.05, ** denotes *p* < 0.01, and *** denotes *p* < 0.001.

Encouraged by the synergistic antitumor effect that was recorded in vitro, we further investigated the role of the CSP nanoparticles in a xenograft tumor mouse model. Tumor-bearing BALB/c nude mice were randomly categorized into six groups and administered with various treatments, including PBS, Cu_2−x_Se@PEG, Cu_2−x_Se@PEG plus laser, SOR, CSP, and CSP plus laser (Figure 6a). Without laser irradiation, the administration of PBS, Cu_2−x_Se@PEG, or CSP alone had no appreciable inhibitory effect on the growth of the tumors, further indicating that the CSP nanoparticles had good biocompatibility. However, the treatment with CSP that was coupled with laser irradiation presented a significant inhibitory effect on tumor growth (Figure 6b,c). Furthermore, the volume and weight of the tumors were also measured. The mean tumor volume in the CSP plus laser group was the smallest among all of the groups (Figure 6d). Meanwhile, no apparent difference was observed between the control PBS and Cu_2−x_Se@PEG groups (Figure 6e). On the contrary, the mean weight of the tumors in the CSP plus laser group was significantly lighter than that of Cu_2−x_Se@PEG plus laser or SOR alone. Subsequently, the pathomorphological assay was employed in order to assess the treatments’ tumor-killing efficacies in different groups by hematoxylin and eosin staining. No obvious damage was spotted in the mice that were treated with PBS, Cu_2−x_Se@PEG, or CSP, while only a partial region of the tumors was destroyed in the mice that were treated with Cu_2−x_Se@PEG plus laser or SOR alone (Figure 6f). In addition, more nuclear pyknosis and cell shape changes were noted in the CSP plus laser group, further indicating that the synergistic effect of the CSP nanoparticles plus laser irradiation had remarkable advantages in tumor therapy.

Following consideration that cell proliferation and angiogenesis are essential for HCC development and progression [47], the corresponding biomarkers, including Ki-67 and VEGFA, were then examined using immunostaining. The results revealed that the expression levels of Ki-67 and VEGFA in the CSP plus laser group were evidently lower than those of other groups, illustrating that the synergistic effects that were produced by the CSP plus laser group could alter the tumor microenvironment through abrogating HCC cells’ proliferation and angiogenesis (Figure 6g). Based on the results that are mentioned above, we conclude that the CSP nanoparticles that were accumulated in the tumor sites could effectively convert the light into heat and enhance the synergistic effect of the coalesced photothermal and molecular targeted therapy in order to kill the tumor cells during laser irradiation.

### 3.7. Biocompatibility Assessment of the Nanoparticle CSP

**Figure 7:**
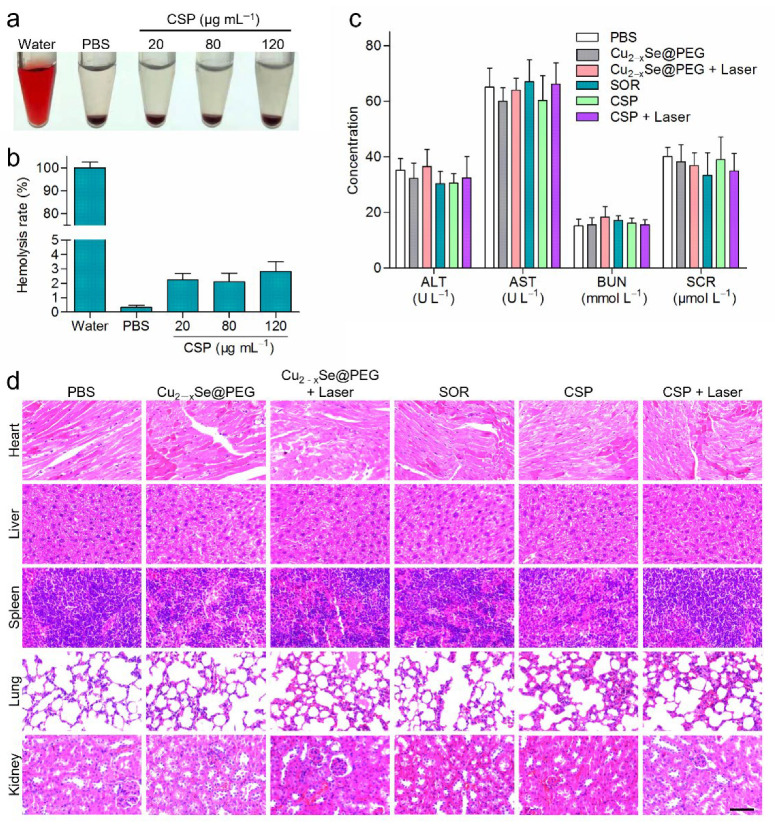
The biocompatibility of CSP nanoparticles. (**a**,**b**) Effect of CSP nanoparticles on the hemolysis of erythrocytes and its comparative analysis. (**c**) Effect of different formulations on liver and kidney functions in a mouse model. (**d**) HE staining of heart, liver, spleen, lung, and kidney of mice after 10 days of various treatments. Scale bar, 50 μm.

Given that the biosafety of nanomaterials is a prerequisite for their biomedical applications, we also evaluated the hemocompatibility of CSP. Compared with the negative control PBS, the CSP nanoparticles ranging from 20 to 120 μg mL^−1^ did not inflict hemolysis when they were incubated with mice erythrocytes (Figure 7a,b). Concurrently, compared with the water group as a positive control, the hemolysis rate of the CSP nanoparticles, even at concentrations up to 120 μg mL^−1^, was less than 5%, indicating the excellent biocompatibility of the as-synthesized CSP. Besides this, we also examined the parameters of hepatic and renal function in order to assess the toxicity of the nanoparticle CSP to the liver and kidney. As shown in Figure 7c, the liver function indicators, including alanine aminotransferase (ALT) and aspartate aminotransferase (AST), as well as the kidney function indicators, incorporating blood urea nitrogen (BUN) and serum creatinine (SCR), had no obvious discrepancy in the mice that were treated with CSP plus laser when compared with those of all of the other groups. Additionally, the toxicity of the CSP to multiple organs, including the heart, liver, spleen, lung, and kidney, was evaluated in the mice after 10 days of treatment using HE staining. No clear damages or lesions were observed in any of the groups (Figure 7d). Collectively, these results reveal that the CSP nanoparticles have excellent potential for HCC treatment in vivo.

## 4. Conclusions

In this work, we rationally fabricated a bifunctional nanoparticle CSP for HCC treatment. The CSP was highly mono-dispersed in an aqueous solution and its average size was approximately 9 nm. The CSP exhibited an excellent photothermal conversion capacity upon 808 nm laser irradiation and the photothermal effect of the photosensitizer Cu_2−x_Se was significantly enhanced after being coordinated with the targeted drug SOR. Thus, the CSP that was loaded with SOR might debilitate the latter’s toxicity and reinforce its efficacy. The biosafety evaluation confirmed that the resultant CSP had good biocompatibility in vitro and in vivo. The cytotoxicity results illustrated an enhanced therapeutic effect for killing tumor cells by inhibiting HCC cells’ proliferation and metastasis, followed by propelling cell apoptosis. Additionally, our in vivo experiments indicated an outmaneuvering of tumor growth through altering the tumor’s microenvironment in order to abrogate tumor cell proliferation and angiogenesis. This study also provides a simple and efficient modality that may be used to generate Cu_2−x_Se-based theranostic agents for biomedical applications.

## Data Availability

Not applicable.

## Supplementary Materials

The following supporting information can be downloaded at: https://www.mdpi.com/article/10.3390/nano12183191/s1, Figure S1: Elemental analysis of Cu_2−x_Se; Figure S2: XPS of Cu_2−x_Se; Figure S3: Structure of PMHC18-mPEG; Figure S4: The hydrodynamic size of Cu_2−x_Se@PEG; and Figure S5: Photothermal properties of Cu_2−x_Se@PEG.
