# Supplementary material for: Sorafenib-Loaded Cu2−xSe Nanoparticles Boost Photothermal–Synergistic Targeted Therapy against Hepatocellular Carcinoma
Source: Nanomaterials (Basel). 2022 Sep 14;12(18):3191. doi: 10.3390/nano12183191 (PMC9505850; doi:10.3390/nano12183191)
Supplement: Supplementary file 1 [file nanomaterials-12-03191-s001.zip › nanomaterials-1899677-supplementary.pdf]

Sorafenib-Loaded $Cu_{2-x}Se$ Nanoparticles Boost Photothermal–Synergistic Targeted Therapy against Hepatocellular Carcinoma

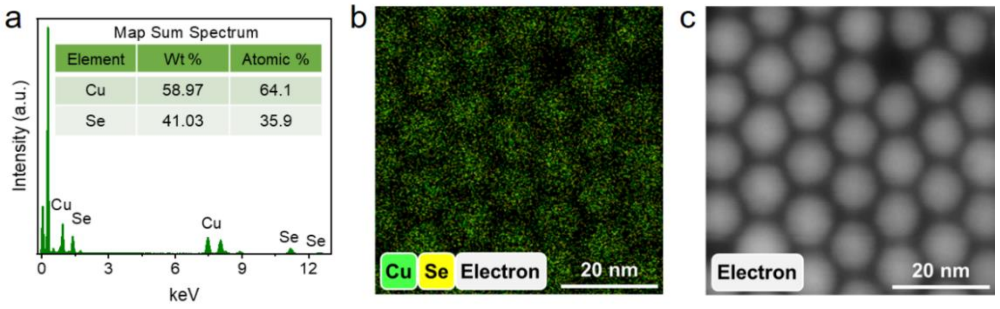

Figure S1. Elemental analysis of $Cu_{2-x}Se$. (a) Energy disperses spectrum (EDS), (b) merged EDS mapping of Cu and Se elements, and (c) HAADF-STEM image.

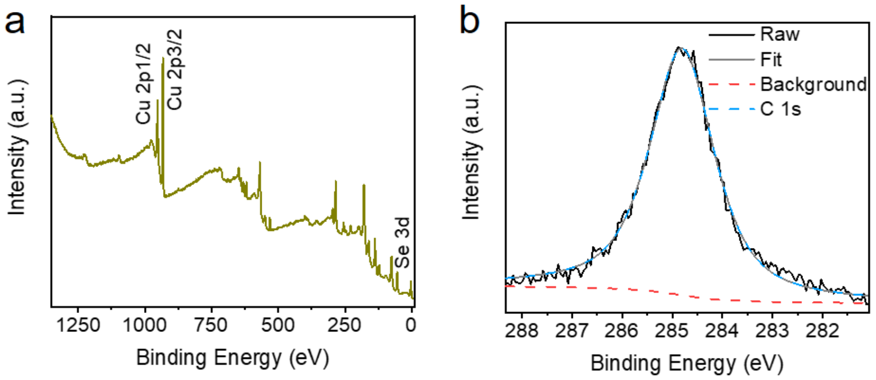

Figure S2. XPS of $Cu_{2-x}Se$. (a) the survey spectrum and (b) the HR-XPS of C 1s.

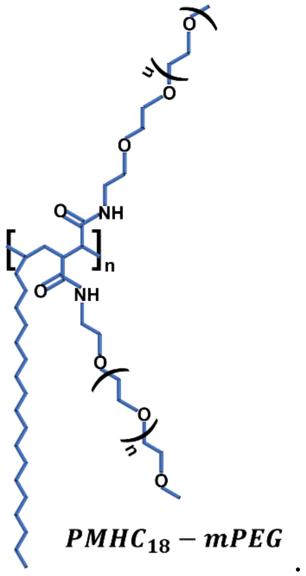

Figure S3. Structure of PMHC18-mPEG.

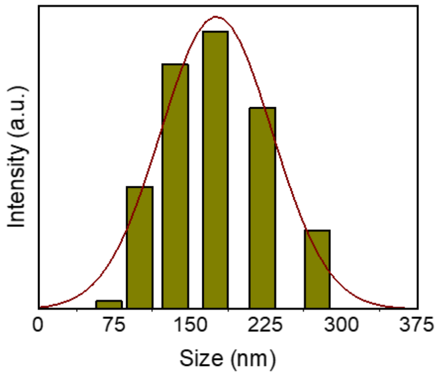

Figure S4. The hydrodynamic size of Cu2-xSe@PEG.

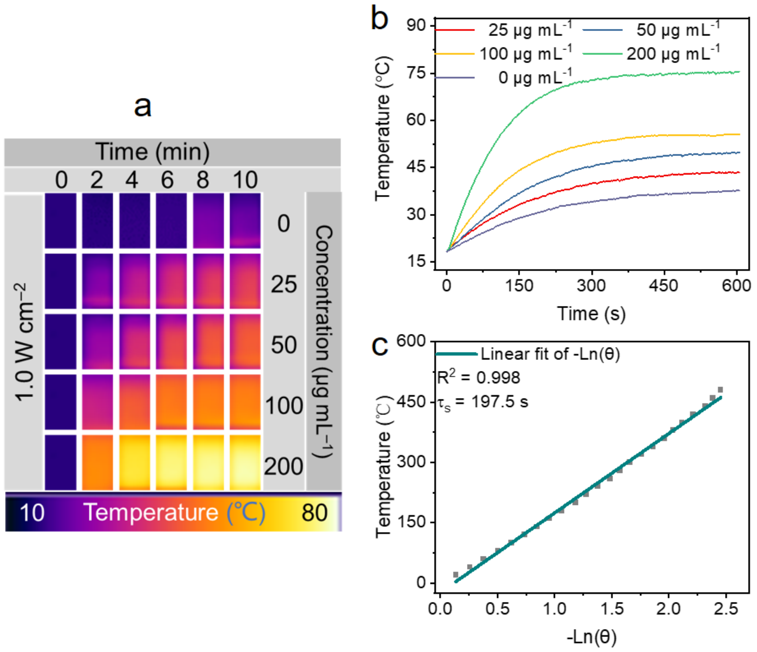

Figure S5. Photothermal properties of Cu2-xSe@PEG excited by laser of 808 nm in water with the laser power of 1.0 W cm^{-2}. (a) Photothermal images with different concentrations, (b) temperature change with increasing concentration, (c) −Ln(***θ***) fitted from Figure. 2e.
